# Neuropathology of AIDS: An Autopsy Review of 284 Cases from Brazil Comparing the Findings Pre- and Post-HAART (Highly Active Antiretroviral Therapy) and Pre- and Postmortem Correlation

**DOI:** 10.1155/2012/186850

**Published:** 2012-02-22

**Authors:** Ana Cristina Araújo Lemos Silva, Blenda Sousa Carli Rodrigues, Adilha Misson Rua Micheletti, Sebastião Tostes, Antonio Carlos Oliveira Meneses, Mário Leon Silva-Vergara, Sheila Jorge Adad

**Affiliations:** ^1^Discipline of Special Pathology, Universidade Federal do Triângulo Mineiro (UFTM), Avenida Getúlio Guaritá 130, 38025-440 Uberaba, MG, Brazil; ^2^Scientific Initiation Scholarship, Conselho Nacional de Desenvolvimento Científico e Tecnológico (CNPq), UFTM, 38025-440 Uberaba, MG, Brazil; ^3^Discipline of Forensic Medicine, UFTM, Avenida Getúlio Guaritá 130, 38025-440 Uberaba, MG, Brazil; ^4^Discipline of Infectious and Parasitic Diseases, UFTM, Avenida Getúlio Guaritá 130, 38025-440 Uberaba, MG, Brazil

## Abstract

A retrospective study of central nervous system (CNS) in 284 autopsy AIDS cases in Brazil (1989–2008) divided into 3 groups: A (without antiretroviral treatment: 163 cases); B (other antiretroviral therapies: 76 cases); C (HAART for 3 months or more: 45 cases). In 165 (58.1%) cases, relevant lesions were found, predominantly infections (54.2%); the most frequent was toxoplasmosis (29.9%) followed by cryptococcosis (15.8%), purulent bacterial infections (3.9%), and HIV encephalitis (2.8%); non-Hodgkin lymphomas occurred in 1.4% and vascular lesions in 1.1%. There was no difference when compared the frequency of lesion among the groups; however, toxoplasmosis was less common while HIV encephalitis was more frequent in group C related to A. CNS lesions remain a frequent cause of death in AIDS; however, the mean survival time was four times greater in group C than in A. In 91 (55.1%) of 165 cases with relevant brain lesions (or 32% of the total 284 cases), there was discordance between *pre-* and *postmortem* diagnosis; disagreement type 1 (important disease that if diagnosed in life could change the patient prognosis) occurred in 49 (53.8%) of 91 discordant cases (17.6% of the total 284) indicating the autopsy importance, even with HAART and advanced diagnostics technologies.

## 1. Introduction

The CNS is the second most-affected organ in AIDS, second only to the lung [1–4]. The immunity deficiency triggered by HIV favors the development of infections by opportunistic agents, being Toxoplasma gondii, Cryptococcus neoformans, and cytomegalovirus the most common in CNS [5–8]; regarding cancer predominates primary non-Hodgkin lymphomas [2, 9]. HIV-specific lesions appear, in general, later in the evolution of the disease, with giant multinucleated cells often found, considered to be “pathognomonic” of HIV infection in CNS [10, 11]. Necropsy is an important method to understand not only the natural course of a disease, but it also enables, through acquired knowledge, a better clinical approach in providing patients with early diagnosis, better management of patients with AIDS, and the election of a prevention option [3, 8]. Necropsy is also a valuable tool for recognizing clinically undiagnosed infections and malignant neoplasms [12–14]. However, systematic studies of autopsy series from HIV-positive patients evaluating the neuropathological findings are infrequent and most have evaluated the pre-HAART period [4, 5, 7, 9, 15–18] and even scarcer are post-HAART studies [2, 3, 19–23], perhaps because there was an important reduction in the number of autopsies from HIV-positive patients [3, 19, 21, 22]. However, they remain important even after HAART, as changes occurred after that therapy, such as a decrease in opportunistic infections and the increase of HIV encephalitis [24], at least in developed countries, although in the rest of the world, in over 90% of AIDS cases, patients do not survive long enough to present HIV neurological complications [25]. However, these autopsy studies are even rarer in developing countries, which makes it difficult to establish the true prevalence of the neuropathological lesions [26]. The importance of these studies can be felt when analyzing the frequencies of some diseases, for example, tuberculosis meningitis, which was seen in 12% of HIV autopsies in India [16], similar frequency to that found in Africa (11%), while in the USA occurred in only 1.4% of cases [27]. Though, we found no comparative study of CNS findings before and after HAART on autopsy series in developing countries (through Medline search).

Among the autopsy series that analyzes changes in the CNS pre- and post-HAART, Masliah et al. [3] compared the frequency of lesions per year for 15 years (1982 to 1998), but they did not separate those cases by groups and do not mention how many did or did not use HAART. It was observed a decrease in opportunistic infections and an increase of HIV encephalitis in the last years of the study. Jellinger et al. [2] and Morgello et al. [20] separated into three periods, according to the therapeutic regimen at the time, but did not evaluate data regarding the use of HAART. Neuenburg et al. [21] and Vago et al. [22] divided the years researched into four periods, the last one corresponding to the HAART period; in the Neuenburg et al. [21] study only 24 (57%) of 42 patients from this last period were using such medication and Vago et al. [22] found 96 (90.6%) of 106 patients using HAART, but do not refer for how long. Gray et al. [19] evaluated 23 cases of autopsies who received HAART for at least 3 months before death, and Walsh et al. [23] evaluated 11 cases in the post-HAART era, however, 2 never used such medication and only 2 (12.5%) used HAART more than 3 months without interrupting the use of more than 2 months before death. This last study, although in a small series, shows that relying only on the time of autopsy, as occurred in most studies, may not reflect the reality on the use of medication. These data are important because there is an initial rapid increase in CD4 counts in the first 3 months of therapy, although still generally increasing more slowly in subsequent years [28], observing a significant improvement of immunity in the first 3–6 months [29], and do not use HAART for more than 2 months characterizes abandonment with loss of the medication benefit [30].

Wilkes et al. [14] highlight the value of autopsy in AIDS to the correlation between pre- and postmortem. From 101 autopsies evaluated, 74% cases of AIDS-related illnesses were not suspected clinically, such as cytomegalovirus (49%), fungal systemic (20%), Kaposi's sarcoma (14%), mycobacteriosis (11%) and CNS lymphoma (4%). Comparison of *pre- *and *postmortem* findings in AIDS was also performed by Antinori et al. [31] in 1630 autopsies, between 1984 and 2002, divided into four periods according to the use of antiretroviral therapy; however, only invasive fungal infections were evaluated. They found invasive fungal infections in 297 cases, which 54.2% were not suspected clinically, with no variation in percentage over the periods. In 38 (36.9%) of 103 cases in which mycosis was the main disease, the diagnosis was not suspected *premortem*, and in 17 (45%) of these 38 cases, the error would be Goldman's type I. This study confirms the value of autopsy in AIDS patients, even in the HAART era.

The objectives of this study were to characterize CNS lesions in AIDS autopsies over 20 years, according to antiretroviral therapy, to assess whether there were changes in the pattern of lesions and in survival time among the study groups and if relevant lesions to death were suspected clinically.

## 2. Material and Methods

This study was approved by the Research Ethics Committee from the Federal University of Triângulo Mineiro (number 831). It is a retrospective study of consecutive autopsies from HIV-positive patients (minimum time of hospitalization 24 hours) performed in a teaching hospital in Uberaba, MG, Brazil, from January 1989 to December 2008. From 1989, we adopted a systematic collection of blood from all autopsy cases, to conduct HIV testing. Clinical reports obtained from medical records and autopsy requests were written in a form, registering all clinical hypotheses made to the CNS, use or not of antiretroviral therapy (type and duration of use), survival time after HIV infection diagnosis, cause of death stated in the autopsy report and morphological data from all organs obtained in the autopsy report, and adding CNS microscopic review, in all cases, by the author of this study. Data concerning CD4 cell count (absent in 16.5% of cases) and viral load (which only began to be routinely performed in our institution from 2000) were not systematically analyzed in the present study, although eventually in existing cases, they were useful for eventual consideration of immunodeficiency.

The standardization sample for microscopy of the CNS in autopsies of our service is a minimum of three tissue blocks with sample from different lobes (one block), basal ganglia and hippocampus (one block), midbrain, pons, and cerebellum (one block); additional blocks were taken from macroscopic lesions. Paraffin sections (5 *μ*m) were stained with hematoxylin-eosin and, where appropriate, special staining techniques were carried out for microorganisms and demyelinating disease assessment (Gram, Grocott, Mucicarmin, Schiff's periodic acid, Fite Faraco, Luxol Fast Blue). Immunohistochemical reaction (polymer technique) was made in some cases using monoclonal antibodies for toxoplasma (Novocastra-NCL-TG-TP3), cytomegalovirus (Novocastra-NCL-CMV-pp65), herpes virus (Dako HSV-I and II), JC virus (Novocastra-NCL-JCBK) and for characterization of lymphoma: CD3 (Dako-CD3-Leu, T3), CD20 (Novocastra, NCL-CD20-L26), CD 30 (Dako-CD30 Ber-H2), CD45 (Dako CD45RO-UCHL1), CD45-LCA (Dako CD45, LCA-2B11 + PD7/26). *In situ* hybridization for Epstein Barr was performed in three cases of non-Hodgkin lymphoma.

The cases were divided into three groups: (A) without any antiretroviral medication; (B) use of 1 or 2 antiretrovirals for any period of time, HAART for less than 3 months or abandoned the use of HAART for more than 2 months; (C) patients on HAART for 3 months or more. During the review of the medical records, it was registered when HIV infection diagnosis was made and the date of death, being calculated the survival time in months. Patients whose diagnosis of HIV infection was made at autopsy or during the hospitalization in which death occurred (hospital stay from 1 to 29 days), the survival time was considered zero. All pathological findings found by CNS microscopic review were tabulated, as mentioned earlier. Then, it was assessed whether findings would be important or not, clinically, not considering relevant focal scarring (focal gliosis, mild mononuclear infiltrate in the meninges, mild ependimite, meningeal fibrous thickening), focal/mild hemorrhage, mild edema, or edema resulting from the final event of the death. Next, where significant pathological findings in the CNS, or with evolutive potential, were found we carried out a comparison with the clinical hypotheses, in order to evaluate the *pre*- and *postmortem* agreement. For those cases with diagnostic disagreements between the anatomopathological lesions in the CNS and the clinical hypotheses, we used the classification proposed by Goldman et al. [32], simplified by de Almeida et al. [12], separating them as follows: type 1 disagreement: an important disease, which clinically diagnosed might had changed the prognosis; type 2 disagreement: important disease not diagnosed, however, if clinically diagnosed, would not have changed the prognosis; type 3 disagreement: minor disease not diagnosed, with doubtful or no influence on prognosis.

The morphological data from different organs, recorded in the autopsy reports, were used to assist in diagnosis of immunodeficiency (such as, opportunistic infections indicative of AIDS, according to the Centers for Disease Control, CDC, USA, 1993) and also to evaluate the immediate cause of death.

## 3. Results

From January 1989 to December 2008, 1785 autopsies were made in our hospital, and 299 (16.7%) cases showed seropositive for HIV. We excluded 15 of 299 cases: 9 for having no criteria for defining AIDS (according to the Centers for Disease Control, CDC, USA, 1993) and 6 by the absence of CNS histological sections or no medical record found; therefore, 284 AIDS autopsies have been used in this study. Ninety of these 284 cases (autopsies from 1989 to 1996) had already been partly previously analyzed in the author's master's thesis [33], corresponding to the period before the introduction of HAART in our Institution. Eighty (28.2%) of 284 cases were female; an increase in the percentage of the female population was observed over the years evaluated, which was 17.8% in the 1989 to 1996 period and increased to 33% from 1997 to 2008. Regarding age, considering a total of 284 cases, the mean age was 35.7 ± 10.8 years (minimum 16 and maximum 69 years). There was a slight increase when comparing the period from 1989 to 1996 (34.3 ± 11.3) to the one from 1997 to 2008 (36.3 ± 10.5). The main risk factors for HIV infection were drug abuse in 33.1% (94 of 284 cases; frequency of 37.2% among 204 male cases and 22.5% among 80 female cases); homosexuality/bisexuality in 14.1% (homosexuality/bisexuality in males 19.6%, while in female patients only 1%). Among 80 female cases, 22.5% reported as a risk factor HIV-positive partner, and also 22.5% reported promiscuity. Several patients reported 2 risk factors and in 27.1% of 284 cases there was no reference about the risk factor.

The percentage of cases with drug use was slightly lower in the period 1989–1996 in comparison to that from 1997–2008 (35.6% versus 32%); yet regarding homosexuality/bisexuality, until 1996 the frequency was much higher than after 1997 (24.4% versus 9.3%), which likely results, at least partially, of the increase of AIDS cases in women since 1997 (17.8% versus 33%).

Regarding the use of antiretroviral medication, since 1993 some patients were already using monotherapy; however, HAART was introduced in our service from 1997. Although only 90 of the total 284 cases are before 1997, most individuals in this study did not have any antiretroviral treatment, fitting them into group A (163 cases = 57.4%). The large number of patients without use of antiretroviral is probably due, in part, also to the fact that in 69 (24.3%) cases the diagnosis of HIV infection was made at autopsy (18 cases; minimum time of hospital stay 24 hours) or during hospitalization in which death occurred (maximum length of hospital stay 29 days), represented by 17 (18.9%) of 90 cases before 1997, and 52 (26.8%) of 194, from 1997. In all 18 cases, whose diagnosis of HIV/AIDS was made only after death, there were opportunistic infections in CNS (toxoplasmosis or cryptococcosis) or in another organs (cytomegalovirus, pneumocystis pneumonia, disseminated mycobacteriosis, or histoplasmosis). These data demonstrate that HIV patients evaluated in this study often sought late medical attention. Of 284 cases, 75 were treated with HAART for some time; however, 30 of these patients had used HAART for less than 3 months or abandoned the treatment for more than 2 months before death. These 30 patients, added to 46 patients who used only one or two antiretroviral constituted group B (76 cases, 26.8%). Then in group C were only 45 (15.8%) cases.

### 3.1. Characterization and Frequency of Morphological Findings in the CNS According to the Study Groups

In 165 (58.1%) of 284—cases relevant brain lesions were identified. Throughout the period and studied groups, infectious lesions (54.2%) greatly predominated in relation to neoplasms (2.5%) and vascular lesions (1.1%). The result of these data is presented in Table 1.

As shown in Table 1, toxoplasmosis was the most frequent infection, seen in 85 (29.9%) of 284 cases, followed by cryptococcosis in 45 (15.8%) cases, purulent bacterial infections in 11 (3.9%) cases, HIV encephalitis in eight (2.8%), and cytomegalovirus infection in 7 (2.5%) cases. Regarding noninfectious lesions, which occurred only in 10 (3.5%) cases, 7 (2.5%) primitive CNS neoplasms and 3 (1.1%) vascular lesions were identified. Among the tumors, there were 2 benign (Grade I meningiomas—World Health Organization) and 5 lymphoid malignancies. These were represented by a case of meningeal plasmablastic plasmacytoma and 4 (1.4%) non-Hodgkin's lymphoma, diffuse type of B cells, associated with Epstein Barr virus infection in all 3 cases this agent was investigated by *in situ* hybridization. Vascular lesions were represented by a case of subarachnoid hemorrhage in brainstem, a case of acute subdural hematoma and a case of massive intraparenchymal hemorrhage. There was no difference when compared the frequency of lesion among the groups; however, toxoplasmosis was less common while HIV encephalitis was more frequent in group C related to A. 

 A comparison of neuropathological lesions in 94 drug abusers and 190 nondrug users showed that bacterial infections (including mycobacteria) were more frequent in drug abusers (9.6% versus 2.6%, Fisher's exact test, *P* = 0.01). There was no statistically significant difference in relation to other lesions.

### 3.2. Analysis of the Relationship between Survival Time after Diagnosis of HIV Infection and Use or not HAART

In 261 (90.9%) of 284 cases there were data on medical records to measure the survival time after diagnosis of HIV infection.  Table 2 shows this result, according to the study groups.

As shown in Table 2, the mean survival time in group C (49.1 months) was more than four times that in group A (11.2 months) and almost twice as long in group B (25.8 months), showing clear benefit of HAART. 

### 3.3. Analysis if the Morphological Lesions in the CNS (*Postmortem*) were Clinically Suspected or not (*Premortem*)

Disagreement was found between the clinical suspicion (*premortem*) and neuropathological findings at autopsy (*postmortem*) in 91 (55.1%) of 165 cases in which significant lesions in the CNS were observed, which represents 32% compared to the total of 284 cases. In 49 (53.8%) of these 91 cases, which represent 17.2% of the total of 284 cases, the disagreement was type 1, that is, important lesion was not clinically diagnosed (*premortem*), which could alter the prognosis; this occurred in all lymphoma cases. Regarding the time, the type I error was 62.2% (23 of 37 discordant cases with significant CNS lesions) until 1996, then decreasing to 48.8% (26 of 54 discordant cases), probably partly due to knowledge acquired through the study of the autopsies that were reported through anatomoclinical sessions weekly. 

In 22 (24.2%) of 91 cases, the disagreement was type 2, that is, important lesions were not diagnosed clinically (*premortem*), but even if they did would not change the prognosis; this occurred in several cases of cerebral toxoplasmosis of patients who died due to lung infections by other agents. In 20 (22%) of 91 cases, disagreement was type 3, that is, less important lesions were not diagnosed clinically (*premortem*), with doubtful or no influence on prognosis; as an example, we could name meningioma in a patient with respiratory failure due to pneumocystis pneumonia. 

## 4. Discussion

Although Brazil is one of the countries with the largest number of AIDS cases (about 600,000 patients) out of Africa, a slightly smaller number than the one estimated for the entire Central and Western Europe [34], this is the first Brazilian study on AIDS autopsy (and also in developing countries) to compare the neuropathological lesions pre-and post-HAART era and to demonstrate that these lesions are still frequent and are often not diagnosed clinically (*premortem*). Necropsy is an important tool to understand the lesions in AIDS and several other diseases. However, there has been an increasing reduction in the number of AIDS autopsy, both in our material as in several other studies [19, 21, 22], probably because the complementary diagnostic and clinical experience have contributed to better management of patients and in obtaining *premortem* diagnosis. 

The percentage of women in our cases has increased over the course of years (from 17.8% up to 1996 to 33% after 1996), following the world trend as also observed by Jellinger et al. [2], whose frequency rose from 7.9% to 29.6%, explaining the differences regarding older studies, such as the Brazilian pre-HAART [9, 18]. The mean age of AIDS patients in this study (35.7 years), which was lower than in developed countries ranging from 38.4 to 40.5 years [2, 3, 21], probably because survival rate is higher in these countries. 

As for the overall frequency of neuropathological findings, we found that in 58.1% of our 284 cases there were significant morphological lesion; a higher value than that found in the Brazilian clinical study De Oliveira et al. [35], which was 46.5%. This difference probably results from greater easiness to diagnose lesions at the anatomopathological study. However, our percentage was lower than the values obtained by Masliah et al. [3], Morgello et al. [20], Walsh et al. [23], Vago et al. [22], and Neuenburg et al. [21], which ranged from 63% to 85%. However, if we compute in our study all cases where there was some kind of CNS lesion (relevant or focal lesions and just edema), as some authors, our percentage would be 94.4%, similar to that found by Chimelli et al. [9] in the pre-HAART era, which was 91.3%. 

Table 3 shows a comparative summary of various series of autopsies studies pre- and post-HAART, regarding to neuropathological lesions. 

Assessing the frequency of all neurological lesions relevant in each group studied, a slightly higher percentage was found in the group without antiretroviral treatment (63.8%) than in the other groups (48.7% and 53.3%), although these differences were not statistically significant. This result could give a false impression that HAART did not alter the prognosis in AIDS; however, the survival time of patients clearly shows benefit of this therapy, although in the final stage, the cause of death is often by neuropathological lesions. Our percentage of lesions in groups was smaller than those of Jellinger et al. [2], which decreased from 80% to 60%. It is possible this higher percentage elapses from the larger CNS sample assessed by these authors, or from not relevant lesions that might have been also computed. Unfortunately, in our study the number of cases of group C was small, perhaps indicating low adherence to HAART in our midst, or possibly because using these drugs makes lower mortality rates, resulting in fewer autopsies, as occurred in several post-HAART studies [2, 19–22]. On the other hand, when we began this study we thought that since 1997 almost all patients would be under regular use of HAART protocol, mainly because these medications are free in Brazil; however, only 23.2% of 194 cases of this period used HAART for 3 months or more and 44.8% of these individuals did not use any antiretroviral medication. These data are difficult to compare because many studies separated groups only for period, without reviewing the clinical data regarding to antiretroviral therapy (Table 3). Vago et al. [22] reported to have collected data related to treatment only in the 4th period: 90.6% used HAART, but the length of treatment was not detailed. If we consider all patients who used HAART for any time, they constitute 38.7% (75 of 194) of the autopsies since 1997; however, considering the minimum duration of 3 months for the effectiveness of therapy, these cases represent only 23.2% (45 of 194) of this period. 

Regarding the type of lesion found, infections prevailed in all our study groups, diagnosed in 54.2% of 284 cases, much lower than that of Neuenburg et al. [21] and Vago et al. [22], which were 85% and 76%, respectively; difference found perhaps due to more detailed sampling of the CNS, the routine use of immunohistochemistry for various viruses or regional differences. Considering the subtypes of infection, toxoplasmosis was the most frequent in our study as a whole (29.9%). Unlike Masliah et al. [3], Neuenburg et al. [21] and Walsh et al. [23], whose global percentage of toxoplasmosis ranged from 2.8% to 18.7% and where HIV infections prevailed, it also occurred in pre-HAART studies from Anders et al. [5], Petito et al. [17], and Budka et al. [15], whose toxoplasmosis frequencies ranged from 6.7% to 17% and cytomegalovirus and HIV encephalitis predominated. These differences are likely to be related to environmental, geographic and socioeconomic factors. The pre-HAART Brazilians autopsy studies [9, 18] corroborate these ideas, as toxoplasmosis was the most frequent infection ranging from 21% to 34.1%. This difference between studies in developed countries and ours also persisted in the isolated analysis of the group using HAART. In spite of the statistically significant reduction in frequency of toxoplasmosis among our untreated group, compared to the one in use of HAART (36.2% versus 17.8%), these values were higher than those found in studies from developed countries, in which there was reduction of 24% to 8% [2], from 9.4% to 7.1% [21], and 60% to 0% [23]. The second most common infection in our study was cryptococcosis, present in 15.8% of cases and similar in the three subgroups of this study; it was more frequent in our material, even in the HAART group (15.6%) than in the corresponding groups from Neuenburg et al. [21], Jellinger et al. [2], and Masliah et al. [3], whose percentage ranged from 0 to 5.6%. Those differences could be explained, at least in part, because many patients in our country seek medical help when already presenting with advanced neurological disease, affecting the diagnosis and treatment. Viral infections, which together represent more than 50% of lesions in the CNS studies in developed countries, even in the pre-HAART period [5, 7, 15, 17], were not common in our series, accounting for only 6.7% of 284 cases, while infections by protozoa, fungi and bacteria amounted to 49.3% in our material. HIV encephalitis occurred in 2.8% of total cases in our study, cytomegalovirus in 2.5% and JC virus infection in only 0.3%. This lower percentage of viral infections in relation to other series of autopsies is probably justified due to the lower survival rates of our patients, with not enough time available for the development of HIV neuropathological lesions, complicating prior by opportunistic infections, as our patients usually seek medical treatment in advanced stages of AIDS. As to the comparison of viral infections between the groups, cytomegalovirus infection was more frequent in our group without antiretroviral drugs (3.1%), while in the HAART group there was predominance of HIV encephalitis (11.1%). This change is probably related to the longer survival of patients on HAART, as it has already been observed in developed countries for several years. However, the percentage of lesion by HIV was much lower, even in our HAART group, than in other series post-HAART [2, 3, 19–23], in which it varied between 15% and 59.5%. In some of these studies there was a slight reduction in HIV CNS infection over the periods studied [19, 22, 23], unlike others who showed an increase [3, 21], but as mild or moderate forms. Probably the lowest frequency of HIV lesion in our HAART group, is due in part to the fact that immunohistochemistry was not performed in our study, and HIV encephalitis diagnosis was made by the direct visualization of multinucleated giant cells, unlike several of these studies that performed routine immunohistochemical as Neuenburg et al. [21], whose percentage of lesion by HIV in HAART group was 59.5%. In addition to possible socioeconomic and geographic factors, in our material there was still a high frequency of toxoplasmosis and cryptococcosis, which may have slightly reduced the survival of this group of patients. Rare infections in the CNS as paracoccidioidomycosis, sporotrichosis, infection by free-living amoeba (*Balamuthia mandrilares*), were not reported in other studies and may be related to environmental and regional factors. 

 Noninfectious lesions occurred in 3.5% of our cases, also no significant difference between groups (3.7%, 2.6%, and 4.4%); 1.4% represented by primary CNS lymphomas and none of these cases was diagnosed *premortem*. In the series of Jellinger et al. [2] the frequency of this neoplasm was much higher in all time periods, ranging between 6% and 11.2%, as in others post-HAART studies, which ranged from 2.4% to 18.2% [19–21, 23]. Probably because survival is higher in developed countries and frequency of opportunistic infections, lower. Important vascular lesions occurred in 4.4% of our HAART group, having been described in 10% in a corresponding group from Jellinger et al. [2] and 19.1% in the pre-HAART study from Anders et al. [5]. They were infrequent in our study, occurred only in groups B and C, and could not be directly related to AIDS. Such large differences between the various series can be explained, at least in part, by the way we reported those lesions, as in our percentage we considered only the relevant lesions such as extensive bleeding, while Anders et al. [5] reported “bleeding of variable intensity.” Given these data we can conclude that neuropathological lesions, even with the use of HAART, are still common in patients with AIDS, which is consistent with the observations reported by other authors [2, 3, 19, 21, 22]. 

Regarding the analysis of survival time after HIV infection diagnosis and use or not of HAART—which our study clearly demonstrated the benefit of this medication—no detailed assessment was found in the other series of autopsies, but only comments on the study from Jellinger et al. [2] and Neuenburg et al. [21], who stated that survival was increased after HAART. Our data confirm the concepts already well known clinically and are also in agreement with the reduction in the number of autopsies performed on AIDS after the introduction of HAART, although other factors may be involved in this issue, such as the possibility of an earlier-achieved diagnosis of HIV infection. 

For the correlation between clinical and autopsy findings, no similar study was found in autopsies of AIDS in relation to the CNS. Wilkes et al. [14] made a review of 101 autopsies of AIDS in pre-HAART era, comparing *pre*- and *postmortem* diagnoses in various organs; they reported that 74% of cases had lesions not suspected clinically. Another more recent study compared *pre*- and *postmortem* diagnosis in AIDS, although very detailed and classifying errors, assessed only invasive mycoses in different organs. In 36.9% of cases in which this infection was the major disease, this diagnosis was not made clinically [31]. In our series disagreement was observed in 91 (55.1%) of 165 cases with significant lesions in the CNS (or 32% of the total 284 cases). Disagreement type 1 (important disease that if diagnosed in life could change the patient prognosis) occurred in 49 (53.8%) of 91 discordant cases, representing 17.6% of the total 284 cases studied. These data demonstrate that even serious lesions are still not suspected clinically, probably because of clinical similarities and very difficult differential diagnosis, which reinforces the importance of autopsy studies to improve understanding and knowledge of brain lesions, which are common and still prevalent, even with the use HAART and the presence of advanced diagnostic technologies. 

## 5. Conclusions

The present study and other series of autopsies post-HAART have shown that despite beneficial effect of HAART, the lesions in the CNS continue to be frequent and important cause of death in AIDS patients. However, the median survival time in our HAART group was four times higher compared to those without antiretroviral, which has not been evaluated in other studies of autopsies. Toxoplasmosis remained the most frequent CNS infection, despite HAART, but was less common in our HAART group (17.8% versus 36.2%) while HIV encephalitis was more frequent (11.1% versus 1.8%). Nevertheless, the percentage of Brazilian toxoplasmosis is more similar to the pre-HAART studies than the post-HAART from developed countries, probably reflecting geographical or socioeconomic and cultural factors. Disagreement between the pre- and postmortem diagnosis occurred in 91 (55.1%) of 165 cases with relevant brain lesions at autopsy; in 49 (53.8%) of these 91 cases (17.2% of the total 284) was disagreement type 1: it was not diagnosed significant lesion premortem, which could change the prognosis, indicating the importance of the autopsy, even with HAART and with advanced diagnostics technologies. The reduction of type I error percentage (62.2% until 1996 to 48.8% after 1996), may have been at least partly resulting from the knowledge acquired through the autopsy study, which was reported through anatomoclinical sessions weekly. 

## Figures and Tables

**Table 1:** Relevant morphological lesions in the central nervous system in 284 autopsies of patients with acquired immunodeficiency syndrome, according to the study groups.

Type of lesion	Group A		Group B		Group C		Total	
	*n*	**%**	*n*	**%**	*n*	**%**	*n*	**%**
*Infections*	**97**	**59.5%**	**35**	**46%**	**22**	**48.9%**	**154**	**54.2%**
* Protozoa*	**59**	**36.2%**	**19**	**25.0%**	**8**	**17.8%**	**86**	**30.3%**
Toxoplamosis	*59	36.2%	18	23.7%	*8	17.8%	85	29.9%
Amebiasis	0	0	1	1.3%	0	0	1	0.3%
* Fungal*	**30**	**18.4%**	**12**	**15.8%**	**7**	**15.6%**	**49**	**17.2%**
Cryptococosis	27	16.6%	11	14.5%	7	15.6%	45	15.8%
Histoplasmosis	2	1.2%	0	0	0	0	2	0.7%
Paracoccidioidomycosis	1	0.6%	0	0	0	0	1	0.3%
Sporotrichosis	0	0	1	1.3%	0	0	1	0.3%
* Bacterial*	**†8**	**4.9%**	**3**	**3.9%**	**3**	**6.7%**	**†14**	**4.9%**
Meningitis/encephalitis/abscess	5	3.1%	3	3.9%	3	6.7%	11	3.9%
Tuberculosis/mycobacteriosis	4	2.4%	0	0	0	0	4	1.4%
* Virus*	**11**	**6.7%**	**2**	**2.6%**	**6**	**13.3%**	**19**	**6.7%**
Cytomegalovirus	5	3.1%	1	1.3%	1	2.2%	7	2.5%
Nodular encephalitis	2	1.2%	1	1.3%	0	0	3	1.2%
HIV	*3	1.8%	0	0	*5	11.1%	8	2.8%
JC	1	0.6%	0	0	0	0	1	0.3%
*Neoplasms*	**6**	**3.7%**	**1**	**1.3%**	**0**	**0**	**7**	**2.5%**
Lymphoid Neoplasms	4	2.4%	1	1.3%	0	0	5	1.8%
Meningiomas	2	1.2%	0	0	0	0	2	0.7%
*Vascular lesions*	**0**	**0**	**1**	**1.3%**	**2**	**4.4%**	**3**	**1.1%**
Concomitant Lesions	11	3.9%	1	0.4%	2	0.7%	14	4.9%

*Subtotal with relevant lesions*	**104**	**63.8%**	**37**	**48.7%**	**24**	**53.3%**	**165**	**58.1%**

Subtotal normal or without relevants lesions	59	36.2%	39	51.3%	21	46.7%	119	41.9%

*Total*	**163 **	**100%**	**76**	**100%**	**45**	**100%**	**284**	**100%**

Group A: without use of any antiretroviral medication; B: 1 or 2 antiretroviral therapy or HAART for any time less than 3 months; C: HAART for 3 months or more; *P < 0.05; *χ* ^2^ test. Nonrelevant lesions occurred in 103 (36.3%) of 284 cases, represented by focal alterations, scarring or just edema related to the final events; in only 16 (5.6%) cases the brain was completely normal. **†**Among the cases with bacterial infection in group A, one patient showed concomitant infection by coccus and mycobacteria in different regions of the CNS (9 bacterial infections/8 patients).

**Table 2:** Average values of survival time (months) after diagnosis of HIV infection in 261 of 284 autopsies of acquired immunodeficiency syndrome, in which it was possible to obtain this information, according to the study groups.

Group	A	B	C	All cases
	*N* = 145	*N* = 71	*N* = 45	*N* = 261
Mean	11.2	25.8	49.1	**21.7**
Standard deviation	26.1	30.6	30.0	**31.2**
Median	1	11	48	**6**
Min-max value	0–192	0–132	6–144	**0–192**

Group A: without use of any antiretroviral medication; B: 1 or 2 antiretroviral therapy or HAART for any time less than 3 months; C: HAART for 3 months or more; min: minimum, max: maximum. *N*: number of cases it was possible to obtain the survival time. Kruskal-Wallis/Dunn: *P* < 0.001 when comparing all groups.

**Table:** (a)

Pre-HAART	*N*	Les	Tox	Cry	CMV	HIV	PML	Nod Enc	Lym	Vas
Moskowitz et al. 1984 Miami, USA [4]	**52**	**73.0**	**30.8**	**1.9**	**3.8**	**9.6**	**3.8**	**7.7**	**1.9**	**7.6**

Anders et al. 1986 Los Angeles, USA [5]	**89**	**74.0**	**6.7**	**12.4**	**15.7**	**?**	**6.7**	**47.2**	**3.4**	**19.1**

Petito et al. 1986 New York, USA [17]	**153**	**80.0**	**10.0**	**< 3**	**26.0**	**28.0**	**< 3**	**17.0**	**6.0**	**0**

Budka et al. 1987 Europe [15]	**100**	**95.0**	**17.0**	**9.0**	**18.0**	**38.0**	**5.0**	**17.0**	**6.0**	**11.0**

Lang et al .1989 Switzerland [7]	**135**	**88.0**	**26.0**	**4.0**	**10.0**	**16.0**	**7.0**	**13.0**	**7.0**	

Chimelli et al. 1992 Rio/S.Paulo, Brazil [9]	**252**	**91.3**	**34.1**	**13.5**	**7.9**	**10.7**	**0.8**	**6.7**	**3.9**	**7.9**

Wainstein et al. 1992 P. Alegre, Brazil [18]	**138**	**59.0**	**21.0**	**12.0**	**0**	**5.1**	**0**	**0**	**1.4**	**10.0**

**Table:** (b)

Post-HAART	*P*	*N*/ NG	Les	Tox	Cry	CMV	HIV	PML	Nod Enc	Lym	Vas
Masliah et al. 2000 California, USA [3]	**1982–1998**	**390**	**63**	**2.8**	**5.6**	**18.5**	**26.9**	**3.1**	**0**	**9.5**	**0**

Jellinger et al. 2000 Austria [2]	**1984–1999**	**450**									
	1984–1992	190		24.0	3.1	17.4	16.1	5.7	3.1	6.3	8.8
	1993–1995	162	80.0	20.4	4.4	20.4	13.8	8.7	8.1	11.2	6.8
	1996–1999	98	60.0	8.0	4.0	11.0	15.0	5.0	0	6.0	10.0

Morgello et al. 2002 New York, USA [20]	**1979–2000**	**394**	**64.0**				**15.0**	**1.8**		**4.6**	
	1979–1986	88	59.0				10.0	4.0		1.0	
	1987–1995	207	58.0				17.0	0		5.0	
	1996–2000	99	80.0				15.0	2.0		7.0	

Neuenburg et al. 2002 Germany [21]	**1985–1999**	**371**	**85.0**	**7.0**	**1.1**	**25.3**	**37.7**	**3.5**		**9.4**	**12.4**
	1985-1986	32		9.4	3.1	21.9	34.4	6.3		15.6	
	1987–1992	170		7.7	1.2	35.9	33.5	1.2		11.2	
	1993–1995	127		5.5	0.8	15.7	37.0	7.1		7.9	
	1996–1999	42		7.1	0	9.5	59.5	0		2.4	

Vago et al. 2002 Italy [22]	**1984–2000**	**1597**					**30.4**				
	1984–1987	119					54.0				
	1988–1994	1116					32.0				
	1995-1996	256					18.0				
	1997–2000	106					15.0				

Gray et al. 2003 France [19]	**1985–2002**	**343**									
	1985–1991										
	1992–1996										
	1997–2002	23		13.0	0	8.7	17.4	17.4		13.0	

Walsh et al. 2004 Canada [23]	**1985–2002**	**16**	**75.0**	**18.7**	**0**	**0**	**50.0**	**25.0**	**0**	**12.5**	**0**
	1	5		60.0			60.0	20.0		0	
	2	11		0			27.3	27.3		18.2	

Silva et al. (current study) 2011 Uberaba, Brazil [33]	**1989–2008**	**284**	**58.1**	**29.9**	**15.8**	**2.5**	**2.8**	**0.3**	**1.1**	**1.4**	**1.1**
	A	163	63.8	36.2	16.6	3.1	1.8	0.6	1.2	1.8	0
	B	76	48.7	23.7	14.5	1.3	0	0	1.3	1.3	1.3
	C	45	53.3	17.8	15.6	2.2	11.1	0	0	0	4.4

*N*: number of cases, NG: number of cases in each group; *P*: period; the last period corresponds to HAART; Les: lesion; all lesions are expressed in percentages; Tox: toxoplasmosis; Cry: cryptococcosis, CMV: cytomegalovirus, HIV: HIV encephalitis/leukoencephalopathy; PML: progressive multifocal leukoencephalopathy JC virus; Lym: primary central nervous system non-Hodgkin lymphoma; Vas: vascular lesion (infarction, hemorrhage). Only Gray et al. Walsh et al. and the current study separated the groups by analyzing the therapy used in each patient; the others divided the groups according to treatment available at the time.
